# Online Microextraction Coupled with HPLC-ABTS for Rapid Analysis of Antioxidants from the Root of *Polygonum bistorta*

**DOI:** 10.1155/2023/7496848

**Published:** 2023-01-17

**Authors:** Wei-Qi Yang, Zheng-Ming Qian, Meng-Qi Wu, Jian-Li Gao, Qi Huang, Yuan-Sheng Zou, Dan Tang

**Affiliations:** ^1^Key Laboratory of Digital Quality Evaluation of Chinese Materia Medica of State Administration of TCM and Engineering & Technology Research Center for Chinese Materia Medica Quality of Guangdong Province, Guangdong Pharmaceutical University, Guangzhou 510006, China; ^2^Xiangnan University, Chenzhou 423000, China; ^3^Dongguan HEC Cordyceps R&D Co., Ltd., Dongguan 523850, China; ^4^School of Pharmaceutical Sciences, Zhejiang Chinese Medical University, Hangzhou 310053, China

## Abstract

The root of *Polygonum bistorta* (PB) is a traditional Chinese medicinal plant material widely used in China. It has been commonly used for the treatment of hemostasis, detumescence, diarrhea, snake bite, and acute gastroenteritis. However, the research on the antioxidant properties and bioactive compounds from PB is inadequate. In the current research, an online microextraction (OLME) coupled with a high-performance liquid chromatography coupled with the 2,2-nitrogen-di (3-ethyl-benzothiazole-6-sulfonic acid) diammonium salt antioxidant assay (HPLC-ABTS) system for rapid analysis of antioxidants from PB was proposed. The PB sample (0.17 mg) was online extracted by mobile phase (acetonitrile and 0.2% acetic acid); a Poroshell 120 SB-Aq column was used for separation; then, an online ABTS assay system was used for screening the antioxidants. Finally, ten components were found in PB, and among them, eight components possessed antioxidant activities. Furthermore, five components (gallic acid, neochlorogenic acid, caffeic acid, chlorogenic acid, and an unknown compound) were proved as major antioxidants when compared with rutin as an antioxidant marker. The results showed that the developed OLME-HPLC-ABTS system was a simple, rapid, green, and efficient instrument for the screening of antioxidants from PB, which provides a powerful tool for the discovery of natural antioxidants in Chinese medicines.

## 1. Introduction

The root of *Polygonum bistorta* (PB) is a traditional Chinese medicinal plant material, which is widely used in China. It has the effects of hemostatic swelling reduction, treatment of acute gastroenteritis, diarrhea, and venomous snake bites [1]. Chemical studies have demonstrated organic acids, flavonoids, and triterpenoids to be its main constituents [2, 3]. Pharmacological research studies have proved that PB has antioxidant, anti-inflammatory, antibacterial, and anticancer activities [3]. The antioxidant activity is one of the most significant functions of PB because the scavenging process of antioxidants plays an important role in preventing the harmful effects of free radicals. It is well known that oxidative stress is associated with aging, inflammation, and cancer and can cause various chronic diseases including hypertension, diabetes mellitus, aging, and Alzheimer's disease [4, 5]. PB crude extraction has been reported to possess a high antioxidant capacity [6]. However, the antioxidant components of PB are still unclear. Therefore, screening of antioxidants from PB is important for the further development of its healthy products.

Traditional screening methods for natural antioxidants include sample extraction, separation, purification, and bioactivity tests; however, these processes are often time-consuming and laborious [7]. Recently, HPLC combined with bioactivity screening methods such as HPLC-ABTS/DPPH/FRAP has been widely used in the screening and identification of antioxidants from natural products, such as Coptidis Rhizoma, Pu-erh tea, and Eagle tea [8–10], which are more rapid and efficient component screening technologies. However, these methods can only reduce some unnecessary separation steps, and the whole process still requires a lot of labor, time, and organic reagents. Fortunately, the online microextraction coupled with HPLC (OLME-HPLC) analysis technology was developed recently and applied for the screening of antioxidants from Chinese medicines [11, 12]. By using OLME-HPLC analysis technology, the samples were extracted by a mobile phase, and then, the crude extractions were separated by HPLC, which avoided the complex sample extraction, purification, and bioactivity test. Furthermore, it can be conveniently applied to the examination of natural materials or other solid materials, especially for those medicinal materials that are inaccessible or valuable. Compared with the offline systems, OLME also possesses other advantages, such as minimal sample contamination, a higher degree of automation, and higher sensitivity [13].

In the present study, the OLME-HPLC-ABTS method was established for screening and identification of antioxidants from PB. The results showed that ten compounds were found in PB, and eight compounds within them possessed antioxidant activities. Among them, five major antioxidants were further estimated by using rutin as the antioxidant marker, including gallic acid, neochlorogenic acid, caffeic acid, chlorogenic acid, and an unknown compound.

## 2. Materials and Methods

### 2.1. Materials and Reagents

PB was purchased from Anhui Bozhou Traditional Chinese Medicine City (Lot number: QS-2019-02, source: Bozhou, China) and authenticated as the dried roots of PB by one of the authors (Dr. Zhengming Qian). Voucher specimens (HEC-PB-005) were deposited at the Dongguan HEC Cordyceps R&D Co., Ltd. (Dongguan, China).

The caffeic acid (Lot number: 5334) was obtained from the Shanghai Standard Biotech Co., Ltd. (Shanghai, China). Rutin (Lot number: 100080-201811) was purchased from the National Institutes for Food and Drug Control (Beijing, China). Chlorogenic acid (Lot number: A22GB158496), neochlorogenic acid (Lot number: M07GB140938), and gallic acid (Lot number: Y19M8C36143) were provided by the Shanghai Yuanye Biotechnology Co., Ltd. (Shanghai, China).

ABTS (Lot number: 39828), potassium persulfate (Lot number: C2017065), and acetate (HPLC grade; Lot number: B2223309) were obtained from Aladdin (Los Angeles, United States). Acetonitrile (HPLC grade; Lot number: 880501) and methanol (AR grade; Lot number: 2021050802) were supplied by Chengdu Kelong Chemical Co., Ltd. (Sichuan, China). Purified water was prepared with a Milli-Q purification system (Millipore, Billerica, MA, USA). All other chemicals and solvents were ACS reagent grade, unless stated otherwise.

### 2.2. Preparation of PB Solution by Offline Extraction

Accurately weighed 0.25 g sample powder into an extraction bottle and then added 5 mL 50% methanol. Subsequently, the crude powder was extracted for 20 min at 30°C by ultrasonic extraction. The sample solution was filtered through a 0.22 *μ*m filter (Agilent Technologies) before HPLC-MS.

### 2.3. Preparation of PB OLME Pool

**Figure 1:**
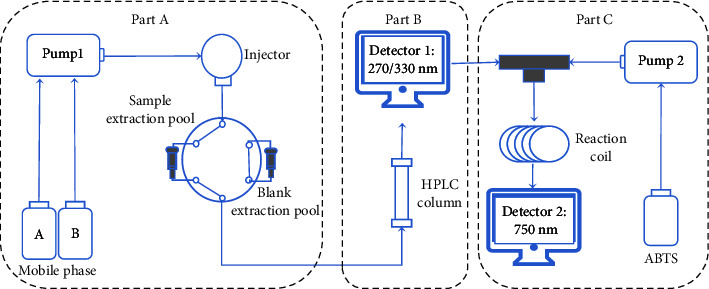
The schematic diagram of the OLME-HPLC-ABTS system.

The dried powder of PB sample was mixed with acid-washed diatomite according to the ratio of 1 : 30. The mixture of PB and acid-washed diatomite powder was weighed accurately (5.0 mg containing 0.17 mg sample) and packed into the hollow guard column (3.0 × 4.0 mm, Phenomenex, Torrance, CA, USA) and then filled up the hollow guard column with acid-washed diatomite. The two ends were sealed with a filter film, respectively. The guard column containing the sample was packed into a matched column holder (Phenomenex). For online system validation by mixed reference solution, the role of the mixture of samples and acid-washed diatomite was replaced by acid-washed diatomite earth. In the guard precolumn sleeve, an online extraction pool is formed and connected to the HPLC system (six-way valve) (Figure 1).

### 2.4. Preparation of Mixed-Standard Solutions

The standards (gallic acid, neochlorogenic acid, caffeic acid, chlorogenic acid, and rutin) were individually dissolved in methanol. Mixed-standard solutions were prepared by mixing the individual reference stock solutions. Then, we obtained a mixed reference solution with final concentrations of gallic acid 109.26 *μ*g/mL, neochlorogenic acid 263.08 *μ*g/mL, chlorogenic acid 98.58 *μ*g/mL, caffeic acid 10.50 *μ*g/mL, and rutin 169.98 *μ*g/mL.

### 2.5. Preparation of ABTS^•+^ Solution

ABTS radical cation (ABTS^•+^) was prepared by mixing potassium persulfate solution (5 mM in water) and ABTS solution (7 mM in water) in equal volume (v/v = 1 : 1) and stored at 4°C in the dark for 12 h. We diluted the solution with ethanol to an absorbance of approximately 1.0 at 750 nm before using [9, 14, 15].

### 2.6. OLME-HPLC-ABTS Radical Scavenging System

The OLME-HPLC-ABTS system (Figure 1) consisted of two pumps (Pump 1, Agilent G1311B pump; Pump 2, WUFENG M1912W050 pump), an injector (Agilent G1329B autosampler), two detectors (Detector 1: Agilent G7117C DAD; Detector 2: Agilent G1315C DAD), an Agilent Poroshell 120 SB-Aq (50 mm × 4.6 mm, 2.7 *μ*m), and a reaction coil (PEEK tubing, 1.5 m × 0.25 mm i.d.). The conditions of OLME-HPLC-ABTS system were as follows:

  (Part A) OLME: Sample was extracted using an online sample extraction pool. Extraction solvent was the mobile phase at a flow rate 0.8 mL/min with 35°C. The online blank extraction pool was used to equilibrate the HPLC system with the initial proportion of mobile phase, while the online sample extraction pool was connected by a six-way value to the flow path of the OLME-HPLC analysis. The extraction process was triggered by an injection of 3 *μ*L solution.
  (Part B) HPLC separation: An Agilent Poroshell 120 SB-Aq (50 mm × 4.6 mm, 2.7 *μ*m) was used for the sample separation. Solvent A is 0.2% acetic acid and solvent B is acetonitrile. Changes in gradient: 0 min, 97% A and 3% B; 5 min, 97% A and 3% B; 11 min, 87% A and 13% B; 22 min, 80% A and 20% B. The flow rate was 0.8 mL/min with the column temperature being maintained at 25°C. The detection wavelength was set at 270 nm (0–4 min) and 330 nm (4–22 min). The injection volume was 3 *μ*L.
  (Part C) Online antioxidant analysis: After the samples were separated by HPLC, they were mixed with ABTS solution (0.4 min/mL), pumped by pump 2, and reacted in the reaction coil (PEEK tubing, 1.5 m × 0.25 mm i.d.), and then, we detected the antioxidant activity peaks at 750 nm.

### 2.7. HPLC-MS Condition

An offline extraction solution was prepared according to “Section 2.2” for HPLC-MS analysis. The chromatographic conditions were the same as that of the OMLE-HPLC system. HPLC-MS conditions: it was monitored by using an Agilent 6530 Q-TOF LC/MS (Palo Alto, CA, USA) equipped with an electrospray ionization (ESI) source operating in both positive and negative ions and an Agilent DAD detector (Santa Clara, CA, USA); dry gas (N_2_) flow of 8 L; dry gas temperature of 350°C; nebulizer of 38 psi; sheath gas flow rate of 11 min/L; capillary voltage of 3.5 kV; nozzle voltage of 1 kV. Collision energy was set at 10 V, 20 V, and 40 V, respectively.

### 2.8. Antioxidant Activity Assessment

Evaluation of the antioxidant activity of components from PB with negative peaks (UV detector at 750 nm) was performed by the developed OLME-HPLC-ABTS method. We prepared an appropriate mass (mg) gradient of rutin for the construction of calibration curves. The calibration curves were constructed by plotting the negative peak areas versus the mass of rutin. Five masses of rutin were used, and their regression equations were calculated in the form of *y* = *ax* + *b*, where *y* and *x* represented the values of negative peak area in 750 nm and the mass of rutin, respectively.

### 2.9. Method Validation

Specificity validation of the qualitative analysis method was carried out according to the ICH (Q2) guidelines [16]. In the current study, the blank solution, standard solution, and sample solution were performed for the specificity test [12]. Meanwhile, to verify the repeatability, PB samples were prepared and analyzed by the OLEM-HPLC method in triplicate. The relative standard deviation (RSD, %) of the peak area was used as the repeatability measurement standard [10].

## 3. Results and Discussion

### 3.1. OLME-HPLC System Optimization

**Figure 2:**
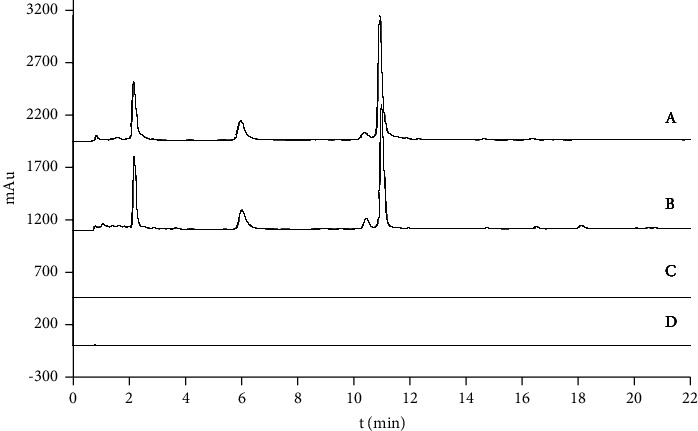
The chromatograms of the OLME-HPLC of PB, the offline sample (A), the OLME sample (B), the secondary OLME sample (C), and the blank sample (D).

To compare the extraction efficiencies, offline sample preparation and online sample extraction were carried out according to the methods under “2.2.1” and “2.2.2,” respectively. The results showed that the peaks obtained by OLME were consistent with the offline extraction peaks (Figures 2 (A) and 2 (B)). Additionally, among them, the PB sample was extracted twice in the online extraction pool under the same conditions. Owing to the fact that the chromatogram of the second extraction was stable and no residual peak was detected (Figure 2 (C)), it was shown that the sample could be completely extracted in the first online extraction. The high extraction efficiency could be attributed to the high pressure of the OLME system, which allows better access of the mobile phase inside the matrix and ensures better extraction [17, 18]. The method is green and efficient, with minimal sample consumption, and can be easily applied to the analysis of natural products or other solid materials, especially for those samples that are difficult to obtain or more valuable.

Organic acids and flavonoids are mainly phenolic components of PB, which had different ultraviolet absorption, and they were generally detected at different wavelengths [19]. Tests were performed at different detection wavelengths (254, 270, 330, and 360 nm) to select the best detection wavelength for the compounds contained in samples. It showed that PB was mainly composed of gallic acid before 4 minutes and had better shape at 270 nm. After 4 minutes, the main components were flavonoids, detected at 330 nm, and the peak distribution was good. So, the final experiential condition in present experiment was the variable wavelength (0–4 min, 270 nm; 4–22 min, 330 nm.).

### 3.2. Method Validation

**Figure 3:**
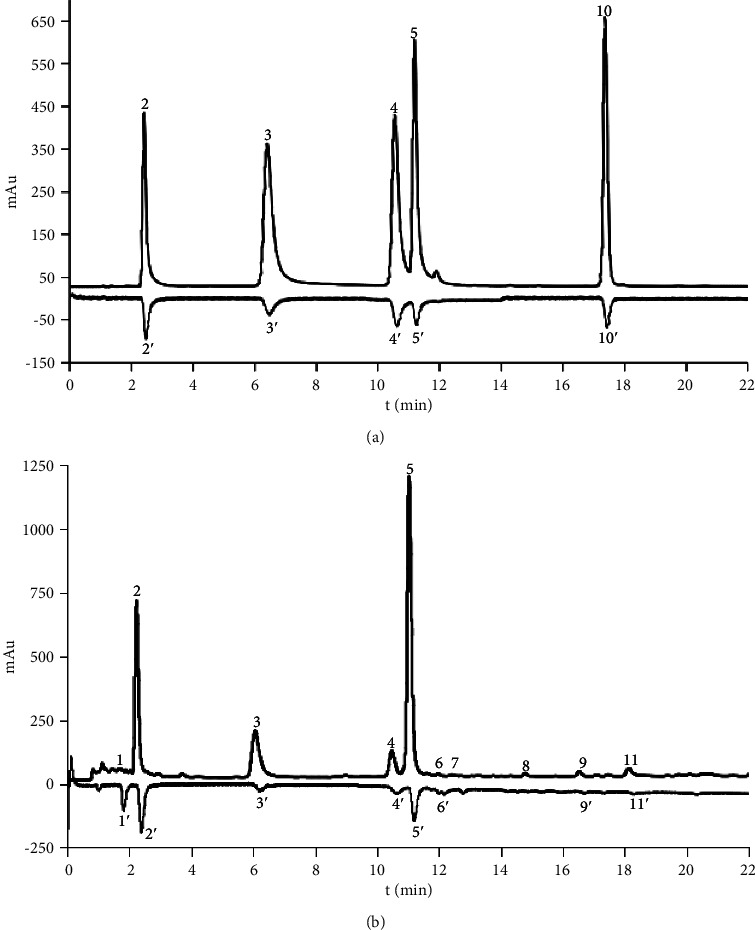
HPLC-chromatograms (positive peaks for components, negative peaks for antioxidants) of mixed standards (a) and PB (b). Unknown (1), gallic acid (2), neochlorogenic acid (3), caffeic acid (4), chlorogenic acid (5), procyanidin B2 (6), catechin (7), quercetin-8-C-glucoside (8), quercetin-4′-O-glucoside (9), rutin (10), and quercetin-3-O-glucuronide (11).

The specificity test results showed that there were no interfering peaks in the online blank chromatogram and the reference material chromatographic peak appears at the corresponding position of the PB chromatogram (Figures 2 (D), 3(a), and 3(b)). The method is based on the repeatability of the peak area in terms of RSD values. They were less than 10%. The results show that the current method was suitable for the analysis of the PB sample.

### 3.3. Identification of Components from PB

The HPLC-MS technique was used to identify the components of PB. Ten peaks were found and nine peaks were identified by comparing the literature data or MS data with the standards. The identification results of components in PB are listed in Table 1.

**Figure 4:**
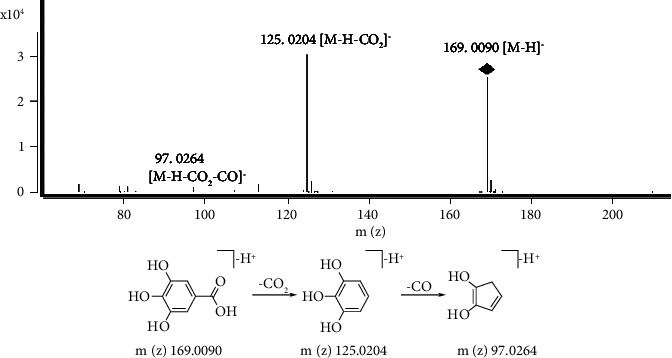
The MS spectrum and major fragmentation pathways of gallic acid.

Identification of organic acids from PB: Organic acids are a type of polyphenolic compounds with their carboxyl groups in the molecular structure. Some common fragmentations occurred by loss of CO, H_2_O, and CO_2_. The excimer ion of peak 2 is m/z 169.0090 [M-H]^−^, with the molecular formula C_7_H_6_O_5_, and the characteristic fragment ion includes 125.0204 [M-H-CO_2_]^−^ and 97.0264 [M-H-CO-CO_2_]^−^. Thus, peak 2 was inferred to be gallic acid (Figure 4). By comparing the MS data and the retention time of chromatographic peaks with standards, it was identified that peaks 3–5 were neochlorogenic acid (peak 3), caffeic acid (peak 4), and chlorogenic acid (peak 5), respectively. The neochlorogenic acid and chlorogenic acid are isomers, which is difficult to differentiate only by MS excimer ion. In order to differentiate them in future research, the retention time and MS fragment were compared. The retention time of neochlorogenic acid is shorter than chlorogenic acid, and the fragment ion (m/z 179) intensity of neochlorogenic acid is higher than chlorogenic acid. These results were complied with the literature [20, 21].

**Figure 5:**
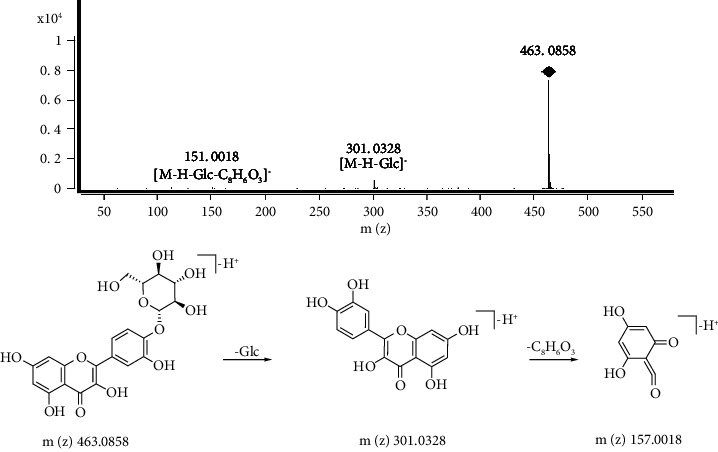
The MS spectrum and major fragmentation pathways of quercetin-4′-O-glucoside.

Flavonoids chemical structures are based on a C6-C3-C6 skeleton. Flavonoids can be directly modified into the carbon atom of the flavonoid skeleton by hydroxylation, methylhydroxylation, C-glycosylation, and O-glycosylation [22]. The excimer ion of peak 9 is m/z 463.0858 [M-H]^−^, with the formula C_21_H_20_O_12_, and the characteristic fragment ions were the ion peaks 301.0328 [M-H-Glc]^−^ and 151.0018 [M-H-Glc-C_8_H_6_O_3_]^−^. Thus, peak 9 was inferred to be quercetin-4′-O-glucoside (Figure 5) [23]. Comparison of the chromatographic peak retention time and the MS data with references [24–26], peaks 6–8 and 11 were identified as procyanidin B2, catechin, quercetin-8-C-glucoside, and quercetin-3-O-glucuronide, respectively. Peak 1 was still unknown based on the present data.

### 3.4. Analysis of Antioxidant in PB

Evaluation of the antioxidant activity of components from PB was performed by the developed OLME-HPLC-ABTS method. ABTS^•+^ is a specie of stable radical which is often used in the evaluation of the general radical scavenging abilities of antioxidants (both pure substances and complex samples). After the sample was mixed with the ABTS solution from post-column instrument, the components with antioxidant activity will react with the free radical cation produced by ABTS solution, which formed an inverted peak in the chromatogram. Comparatively, this method can distinguish the contributions of a single antioxidant compound to the complex samples unlike traditional methods. In this study, eight negative peaks were detected by ABTS assay, which indicated that these components were the antioxidant components from PB (Figure 3), including unknown compound (peak 1′), gallic acid (peak 2′), neochlorogenic acid (peak 3′), caffeic acid (peak 4′), chlorogenic acid (peak 5′), procyanidin B2 (peak 6′), quercetin-4′-O-glucoside (peak 9′), and quercetin-3-O-glucuronide (peak 11′).

Under the chromatographic conditions mentioned above, the calibration curve of *y* = 900.93 *x* + 191.54 exhibited good linear regressions (*R*^2^ = 0.9994) in a relatively wide concentration range. Five major negative peaks (1′–5′) were up to quantify. The other 3 negative peaks (6′, 9′, and 11′) were too low in content and were not up to quantify. The antioxidant activities of 5 negative peaks were estimated by the calibration curve. The results are summarized in Table 1. Gallic acid (peak 2′, 10.98 ± 0.13 mg RE/g) and chlorogenic acid (peak 5′, 8.71 ± 0.60 mg RE/g) were the major antioxidant compounds, and their total contributions of antioxidant activity were up to 55%. The other 3 peaks, namely, peak 1′ (unknown compound), peak 3′ (neochlorogenic acid), and peak 4′ (caffeic acid) with negative peak areas, presented antioxidant activity as 2.52 ± 0.34 mg RE/g (9.46% of total antioxidant activity), 1.85 ± 0.08 mg RE/g (7.73% of total antioxidant activity), and 1.74 ± 0.07 mg RE/g (7.45% of total antioxidant activity), respectively. Gallic acid has been listed in the 2020 edition Chinese Pharmacopoeia as a quality marker of PB [16]. In the current study, we found that chlorogenic acid is important as gallic acid, which should be selected as the quality control marker of PB.

### 3.5. Comparison of Developed and Reported Methods

The comparison of sample extraction and chromatographic separation with reported HPLC methods of PB is listed in Table 2. In previous literature, PB samples were prepared by ultrasonic and heated extraction methods. For example, 500 mg PB was ultrasonically extracted and reflux heated with 60 mL of 20% methanol for 90 minutes [2]. 10,000 mg PB was extracted twice by heating with 100 mL 50% methanol and 100 mL 80% methanol, respectively [27]. The reported extraction methods took over 25 mL solvent, costed over 200 mg samples, and needed 30–120 minutes for sample preparation. In the current study, the sample consumption was only 0.17 mg, and the sample preparation time and solvent consumption were combined with the HPLC separation.

The chromatographic separation was also important in HPLC analysis. Commonly used C18 column for separation of components from PB and the separation time was usually over 40 min [1, 2, 27, 28]. To obtain a rapid separation, the Poroshell column was selected in the current experiment. The separation time of the sample solution was less than 25 min.

## 4. Conclusion

In the current study, antioxidants from PB were rapidly screened and identified by the developed OLME-HPLC-ABTS method. The experimental results showed that 10 compounds were found from PB, and 8 compounds within them possessed antioxidant activities. Among them, five major antioxidants were estimated by using rutin as the antioxidant marker, including gallic acid, neochlorogenic acid, caffeic acid, chlorogenic acid, and an unknown compound. The analysis method also provides the possibility of determining the antioxidant activity of unknown antioxidants to accurately assess the antioxidant activity of known compounds, their amount, and their contribution to the total antioxidant activity of the herbal material. In addition, the developed method is more suitable for the analysis of active substances in valuable medicinal materials due to the low sample consumption (mg level). To conclude, as an eco-friendly analytical chemistry method with little pollution, it provided a possible developmental direction for the discovery of bioactivity compounds from traditional Chinese medicines and further quality control research studies.

## Figures and Tables

**Table 1:** MS data of components in PB.

No.	t/min	Formula	Adducts	Fragment	Identification	REAC
1	1.922	C_6_H_10_O_3_	131.0629 [M + H]^+^	113.0596, 89.0232,61.0284	Unknown	2.52 ± 0.34
2	2.133	C_7_H_6_O_5_	169.0090 [M-H]^−^	125.0204, 97.0264, 79.0160	Gallic acid	10.98 ± 0.13
3	5.773	C_16_H_18_O_9_	353.0836 [M-H]^−^	191.0524, 179.0312, 135.0422	Neochlorogenic acid	1.85 ± 0.08
4	10.038	C_9_H_8_O_4_	179.0325 [M-H]^−^	135.0431, 107.0492, 89.0385, 59.0128	Caffeic acid	1.74 ± 0.07
5	11.079	C_16_H_18_O_9_	353.0863 [M-H]^−^	191.0538, 135.0430, 85.0285	Chlorogenic acid	8.71 ± 0.60
6	12.150	C_30_H_26_O_12_	577.1317 [M-H]^−^	425.0384, 407.0730, 289.0694, 125.0221	Procyanidin B2	+
7	12.535	C_15_H_14_O_6_	290.0790 [M-H]^−^	245.0782, 203.0686, 109.0286	Catechin	−
8	15.294	C_21_H_20_O_12_	464.0955 [M + H]^+^	345.0609, 315.0474, 369.0604	Quercetin-8-C-glucoside	−
9	17.007	C_21_H_20_O_12_	463.0858 [M-H]^−^	301.0328, 151.0018	Quercetin-4′-O-glucoside	+
11	18.567	C_21_H_18_O_13_	477.0657 [M-H]^−^	477.0652, 301.0330, 151.0019	Quercetin-3-O-glucuronide	+

+: negative peak was too low to quantify; −: without negative peak.

**Table 2:** The HPLC methods for analysis of PB.

No.	Sample preparation				HPLC separation			Reference
	Method	Sample (mg)	Solvents	Time (min)	Instruments	Time (min)	Total time (min)	
1	Ultrasonic extraction	200	25 mL 20% methanol	40	HPLC	40	80	[1]
2	Ultrasonic extraction and reflux heated extraction	500	60 mL 20% methanol	90	HPLC	95	185	[2]
3	Heated extraction	10000	100 mL 50% methanol and 100 mL 80% methanol	120	HPLC	60	180	[27]
4	Ultrasonic extraction	2000	50 mL 20% methanol	30	HPLC	70	100	[28]
Current study	Online HPLC extraction	0.17	—	—	HPLC	22	22	—

## Data Availability

The data used to support the findings of this study are included within the article.

## References

[1] Liu R., He F. Y., Li L., Li F. M. (2005). HPLC determination of gallic acid and chlorogenic acid in *Polygonum bistorta* L. *Chinese Journal of Pharmaceutical Analysis*.

[2] Intisar A., Kiazolu J. B., Wang Y., Zhang L., Zhang W. (2012). Effect of mobile phase composition and pH on HPLC separation of rhizome of *Polygonum bistorta*. *Journal of Liquid Chromatography & Related Technologies*.

[3] Wang H. N., Huang B. S., Zhan Z. L., Huang L. Q., Liu D. H., Du H. Z. (2020). Latest research progress of chemical constituents and pharmacological activities of *Polygonum bistorta* L. *Modernization of Traditional Chinese Medicine and Materia Materia-World Science and Technology*.

[4] Musazadeh V., Jafarzadeh J., Keramati M. (2021). Flaxseed oil supplementation augments antioxidant capacity and alleviates oxidative stress: a systematic review and meta-analysis of randomized controlled trials. *Evidence-based Complementary and Alternative Medicine*.

[5] Simpson T., Pase M., Stough C. (2015). Bacopa monnieri as an antioxidant therapy to reduce oxidative stress in the aging brain. *Evidence-based Complementary and Alternative Medicine*.

[6] Chang X., Liu Y. X., Kang W. Y. (2009). Antioxidant activity of extracts from *Polygonum bistorta* L. *Fine Chemical Intermediates*.

[7] Wang W. D., Jiao L. J., Tao Y. D. (2019). On-line HPLC-DPPH bioactivity-guided assay for isolated of antioxidative phenylpropanoids from Qinghai-Tibet Plateau medicinal plant *Lancea tibetica*. *Journal of Chromatography B*.

[8] Li S. J., Wang Y. Q. (2018). On-line scavenging activity of Huanglian by HPLC-ABTS-DAD-Q-TOF-MS. *Zhongguo Zhongyao Zazhi*.

[9] Qian Z. M., Guan J., Yang F. Q., Li S. P. (2008). Identification and quantification of free radical scavengers in Pu-erh tea by HPLC-DAD-MS coupled online with 2,2′-Azinobis (3-ethylbenzthiazolinesulfonic acid) diammonium salt assay. *Journal of Agricultural and Food Chemistry*.

[10] Meng Q., Qian Z., Li X. (2012). Free radical scavenging activity of Eagle tea and their flavonoids. *Acta Pharmaceutica Sinica B*.

[11] Qian Z. M., Fang B. W., Chen H. M. (2020). Online liquid microextraction coupled with HPLC-ABTS for rapid screening of natural antioxidants: case study of three different teas. *Journal of Chromatographic Science*.

[12] Fan W. F., Zhou J. Q., Wu Z. (2021). Analysis of antioxidants in *Chrysanthemum indici* flos by online gradient extraction and HPLC-FRAP. *Analytical Methods*.

[13] Tong C., Peng M., Tong R., Ma R., Guo K., Shi S. (2018). Use of an online extraction liquid chromatography quadrupole time-of-flight tandem mass spectrometry method for the characterization of polyphenols in *Citrus paradisi* cv. Changshanhuyu peel. *Journal of Chromatography A*.

[14] Chen L. X., Hu D. J., Lam S. C. (2016). Comparison of antioxidant activities of different parts from snow chrysanthemum (Coreopsis tinctoria Nutt.) and identification of their natural antioxidants using high performance liquid chromatography coupled with diode array detection and mass spectrometry and 2,2′-azinobis(3-ethylbenzthiazoline-sulfonic acid)diammonium salt-based assay. *Journal of Chromatography A*.

[15] Li D., Zhao J., Li S. (2014). High-performance liquid chromatography coupled with post-columndual-bioactivity assay for simultaneous screening of xanthine oxidase inhibitors and free radical scavengers from complex mixture. *Journal of Chromatography A*.

[16] Chinese Pharmacopoeia Commission *Chinese Pharmacopoeia Commission. Pharmacopoeia of the People’s Republic of China*.

[17] Chen Q., Zhou J. Q., Tan G. Y. (2021). Rapid analysis of antioxidant in Hoveniae Semen by online extraction HPLC-ABTS. *Chinese Journal of Pharmaceutical Analysis*.

[18] Qian Z. M., Li C. H., Song Y. L., Zhou M. X., Li W. J. (2019). Rapid determination of adenosine in Cordyceps by online extraction HPLC. *Journal of Chromatographic Science*.

[19] Qian Z. M., Li H. J., Li P., Ren M. T., Tang D. (2007). Simultaneous qualitation and quantification of thirteen bioactive compounds in flos lonicerae by high-performance liquid chromatography with diode array detector and mass spectrometry. *Chemical & Pharmaceutical Bulletin*.

[20] Stobiecki M., Kachlicki P. (2006). Isolation and identification of flavonoids. *The Science Of Flavonoids*.

[21] Wang Y., Wen J., Zheng W. (2015). Simultaneous determination of neochlorogenic acid, chlorogenic acid, cryptochlorogenic acid and geniposide in rat plasma by UPLC‐MS/MS and its application to a pharmacokinetic study after administration of Reduning injection. *Biomedical Chromatography*.

[22] Li J., Wang S. P., Wang Y. Q. (2021). Comparative metabolism study on chlorogenic acid, cryptochlorogenic acid and neochlorogenic acid using UHPLC-Q-TOF MS coupled with network pharmacology. *Chinese Journal of Natural Medicines*.

[23] Riggi E., Avola G., Siracusa L., Ruberto G. (2013). Flavonol content and biometrical traits as a tool for the characterization of “Cipolla di Giarratana”: a traditional Sicilian onion landrace. *Food Chemistry*.

[24] Ying L., Wang D., Du G. (2021). Analysis of bioactive components in the fruit, roots, and leaves of alpinia oxyphylla by UPLC-MS/MS. *Evidence-based Complementary and Alternative Medicine*.

[25] Xiao Y., Hu Z., Yin Z. (2017). Profiling and distribution of metabolites of procyanidin B2 in mice by UPLC-DAD-ESI-IT-TOF- MS^n^ technique. *Frontiers in Pharmacology*.

[26] Klimczak U., Woźniak M., Tomczyk M., Granica S. (2017). Chemical composition of edible aerial parts of meadow bistort, Persicaria bistorta (L.) Samp.). *Food Chemistry*.

[27] Smolarz H. D. (2014). Flavonoid glycosides in nine *Polygonum L*. taxons. *Acta Societatis Botanicorum Poloniae*.

[28] Dong J. M., Cui J., Zhao X. M., Zhao K. J., Ping Z. (2017). Comparative study on HPLC-MS fingerprint of Bistortae Rhizoma prepared slides in 2 different colors. *Journal of Pharmaceutical Analysis*.

